# Molecular Characterization of Two Genes Encoding Novel Ca^2+^-Independent Phospholipase A2s from the Silkworm, *Bombyx mori*

**DOI:** 10.3390/cimb44020054

**Published:** 2022-02-04

**Authors:** Xin Hu, Bili Zhang, Xi Zheng, Haoyan Ji, Kun Feng, Xiaosong Hu, Isma Gul, Muhammad Nadeem Abbas, Hongjuan Cui, Yong Zhu

**Affiliations:** State Key Laboratory of Silkworm Genome Biology, College of Sericulture, Textile and Biomass Sciences, Southwest University, Chongqing 400716, China; huxinusing@163.com (X.H.); a18286002256@163.com (B.Z.); ccstone2019@email.swu.edu.cn (X.Z.); 18303478069@163.com (H.J.); fk18852895031@163.com (K.F.); HXS930815@163.com (X.H.); ismagul@163.com (I.G.); abbasmndr@163.com (M.N.A.)

**Keywords:** silkworm, *iPLA2*, immune response, development

## Abstract

Eicosanoids are crucial downstream signals in the insect immune responses. Phospholipase A2 (PLA2) catalyzes phospholipids, the initial step in eicosanoid biosynthesis. In mammals, the biological roles of *Ca^2+^-independent Phospholipase A2 (iPLA2)* have been extensively studied; however, only a few studies have attempted to explore *iPLA2* functions in insects. In this study, we identified two *iPLA2* genes (designated as *BmiPLA2A* and *BmiPLA2B*) in the silkworm, *Bombyx mori*. *BmiPLA2A* had a 2427 base pair (bp) open reading frame (ORF) that coded for a protein with 808 amino acids. In contrast, *BmiPLA2B* had a 1731 bp ORF that coded for a protein with 576 amino acids. Domain analysis revealed that BmiPLA2A had six ankyrin repeat domains, but BmiPLA2B lacks these domains. *BmiPLA2A* and *BmiPLA2B* were transcribed widely in various tissues and developmental stages with different expression patterns. The administration of 20-hydroxyecdysone increased their expression levels in the epidermis and hemocytes. Furthermore, challenged with virus, fungus, Gram-negative bacteria, and Gram-positive bacteria induced the expression of *BmiPLA2A* and *BmiPLA2B* with variable degrees along with different time points. Our findings imply that *BmiPLA2A* and *BmiPLA2B* may have important biological roles in the development and innate immunity of *B. mori*.

## 1. Introduction

In living organisms, the *PLA2* superfamily is divided into 16 groups [1], which were further classified into three types based on their properties: *Ca^2+^-dependent cellular PLA2* (*cPLA2*), *secretory PLA2* (*sPLA2*), and *Ca^2+^-independent cellular PLA2* (*iPLA2*) [2]. Among *PLA2*, *iPLA2s* have only been found in a few insects. iPLA2s are Ca^2+^-independent enzymes that range in molecular masses from 28 kDa to 146 kDa [1,3]. iPLA2 plays a key role in membrane remodeling [4], AAs release [5], secretion, and other processes in mammals [6], etc. However, there is little research on iPLA2s in insects despite their relevance in various physiological processes. Insect *iPLA2s*, such as *SeiPLA2A* and *SeiPLA2B,* were first discovered in *Spodoptera exigua*, and have been demonstrated to play crucial physiological roles in insect immunity and larval growth [7,8]. IMD/Toll pathways have been shown to regulate these two i*PLA2s*, and immune challenges with different types of pathogens significantly increased their transcription [9]. Otherwise, *PLA2-VIA* mutation in *Drosophila* caused increased sensitivity to oxidative stress, progressive neurodegeneration, and a severely reduced lifespan [10]. These studies show that insect *iPLA2* may play crucial biological roles in development and immunity.

In addition to *iPLA2s*, some *sPLA2s* have been characterized in different insects. sPLA2s are a family of small secreted proteins (14–18 kDa) that typically have 5–8 disulfide bonds [3]. Since their discovery in *Drosophila melanogaster,* sPLA2s have been widely studied in insects [11]. For example, in *Spodoptera exigua*, a lepidopteran insect, one (*Se-sPLA2A)* has been linked to cellular immune response and larval development [12]. In *Maruca vitrata*, another *sPLA2 (Mv-sPLA2)* has been shown to be upregulated in response to immune challenges [13]. Knockdown with respect to expression remarkably reduced hemocyte-spreading behavior, the formation of hemocytic nodules, and larval development, showing that it plays important roles in immunity and development. *sPLA2s* have also been reported in many other insects including *Bactrocera dorsalis* [14], *B. mori* [15], *Ostrinia furnacalis* [16], *Tribolium castaneum* [17], *Rhodnius prolixus* [18], etc. cPLA2s are large cytosolic proteins with a range of sizes (61–114 kDa) and utilize serine for catalysis. In comparison to sPLA2s, Ca^2+^ is not required for the catalysis of cPLA2s, but it is necessary for translocation of the enzyme to intracellular membranes by binding to a C2-domain [3]. However, so far, no *cPLA2* has been found in the genomes of insects.

*B. mori* is an important model insect for biological research, and understanding its physiological processes will be useful in understanding physiological processes in other insects [19]. In the present study, we first reported two *iPLA2* genes from *B. mori* and studied their expression profiles in depth. We also investigated the expression patterns of *iPLA2s* in *B. mori* after immune challenge with various types of pathogens to better understand their biological roles.

## 2. Materials and Methods

### 2.1. Exprimental Animals

*B. mori* larvae were obtained from the State Key Laboratory of Silkworm Genome Biology at Southwest University (Chongqing, China) and cultured in a laboratory incubator as described in previous studies [20,21].

### 2.2. RNA Extraction and cDNA Synthesis

Total RNA was isolated with the TRIzol reagent (Invitrogen, Carlsbad, CA, USA) according to its protocol. Then, the isolated RNA was subjected to 1.0% agarose gel electrophoresis for the integrity inspection, and quality was determined by an ultraviolet spectrophotometer. RNA measuring 2 μg was used to synthesize the first-strand cDNA by using GoScript™ Reverse Transcriptase (Promega, WI, USA) as previously described [22].

### 2.3. Gene Amplification

Two query sequences from *S. frugiperda*, *SeiPLA2A* (GenBank accession number: KJ995815.1) and *SeiPLA2B* (KY575276.1) were used to obtain orthologous genes of *B. mori* through BlastN search in three databases, including SilkDB (https://silkdb.bioinfotoolkits.net/main/species-info/-1, accessed 20 August 2021), SilkBase (silkbase.ab.a.u-tokyo.ac.jp), and NCBI (https://www.ncbi.nlm.nih.gov, accessed 20 August 2021). The open reading frames (ORF) were determined by the online tool ORF Finder (https://ncbiinsights.ncbi.nlm.nih.gov/tag/orffinder/, accessed on 20 August 2021). Subsequently, the nucleotide sequences of the obtained ORFs were confirmed by polymerase chain reaction (PCR) with gene-specific primers (Table 1). The PCR program was executed as follows: an initial denaturation (94 °C for 5 min), followed by 35 cycles of denaturation (94 °C for 30 s), annealing (55 °C for 30 s), and extension (70 °C for 45 °C) and a final extension (70 °C for 10 min). After purification by 1.0% agarose gel electrophoresis and Multifunction DNA Purification Kit (Biomed, China), the PCR products were sequenced by the Beijing Genomics Institute (Beijing, China).

### 2.4. Bioinformatic Analysis

The online tool TMHMM server version 2.0 (http://www.cbs.dtu.dk/services/TMHMM/, accessed on 10 September 2021) and SignalP-5.0 (http://www.cbs.dtu.dk/services/SignalP/, accessed on 10 September 2021) were used for prediction of the transmembrane region and signal peptide, and SMART (http://smart.embl-heidelberg.de/, accessed on 10 September 2021) and NCBI Conserved Domain Search (https://www.ncbi.nlm.nih.gov/Structure/cdd/wrpsb.cgi, accessed on 10 September 2021) were used for functional motifs and conserved domains prediction. The gene structure was analyzed according to SilkBase.

Software Clustal X and Jalview were employed to conduct homologous sequence alignment with the homologous proteins of BmiPLA2A and BmiPLA2B. The phylogenetic trees of PLA2A and PLA2B proteins were constructed by MEGA 7 software based on a Neighbor-joining method with 1000 bootstrapped replicates. All homologous protein sequences of BmiPLA2A and BmiPLA2B were obtained from NCBI.

The 3D structures of BmiPLA2A and BmiPLA2B were predicted by using SWISS-MODEL (http://swissmodel.expasy.org/interactive, accessed on 10 September 2021), and bioinformatics software PyMOL was used to highlight the conserved domains and motifs of the 3D structures.

### 2.5. Quantitative Real-Time PCR (qRT-PCR) Analysis

The specific primers used for qRT-PCR were designed by NCBI Primer-BLAST and are listed in Table 1. qRT-PCR was conducted using GoTaq^®^ qPCR Master Mix (Promega, USA) on a LightCycler 96 (Roche) apparatus. The eif4A gene (Genbank accession number: DQ443290) was used as a reference gene. The relative mRNA expression level was calculated using the 2−ΔΔCt method [23].

### 2.6. Culture of Microbial Pathogens and Pathogen Stimulation

*E. coli* (Gram-negative bacterium, *Escherichia coli, DH5α*) and *S. aureus* (*Staphylococcus aureus*, Gram-positive bacterium) were grown in Luria-Bertani (LB) medium at 37 °C in a shaking (220 rpm) incubator, then washed twice with 1 × PBS, and finally diluted with 1 × PBS to a concentration of 10^8^ cells/mL. *B. bassiana* (*Beauveria bassiana)* was cultured on potato dextrose agar medium at 25 °C for 7 days. Cultured fungal colonies were washed twice with 1 × PBS and finally diluted with 1 × PBS to a 108 cells/mL concentration.

Four different types of microbes were used to test the immune responses of *BmiPLA2A* and *BmiPLA2B* to immune stimulation in two immune-related tissues, hemocytes and fat bodies. Briefly, silkworm larvae at the 5th instar with similar body weight and size were randomly divided into five groups. Pathogens including Gram-negative bacteria *E. coli* (1 × 10^6^ cells), Gram-positive bacteria *S. aureus* (1 × 10^6^ cells), fungus *B. bassiana* (1 × 10^6^ cells), and virus *BmNPV* (*B. mori nucleopolyhedrovirus*, 500 PFU) were injected into hemocoel with a glass capillary, and 1 × PBS was used as a control. Subsequently, hemocytes and fat bodies were collected at different time points (6 h, 12 h, 24 h, and 48 h) post-infection.

### 2.7. 20-Hydroxyecdysone Induction

The effect of 20-hydroxyecdysone on the expression of BmiPLA2A and BmiPLA2B was detected after 20-hydroxyecdysone hemocoel injection. Briefly, 10 μL 20-hydroxyecdysone (1 µg/µL) diluted in 75% ethanol was injected into each larva, and 10 μL 75% ethanol was used as a control. Relative mRNA expression levels of BmiPLA2A and BmiPLA2B were detected by qRT-PCR at different time points (3, 6, 12, and 24 h) after 20-hydroxyecdysone application.

### 2.8. Statistical Analysis

All data from three biologically independent replicates are presented as the means ± SD. The significance of the difference between the two groups was evaluated using Student’s *t*-test. The asterisks indicate the significance was statistically significant or extremely significant (* *p* < 0.05, ** *p* < 0.01 and *** *p* < 0.001).

## 3. Results

### 3.1. Subsection

#### 3.1.1. Molecular Cloning and Amino Acid Sequence Analysis of BmiPLA2 Genes in Silkworm

**Figure 1:**
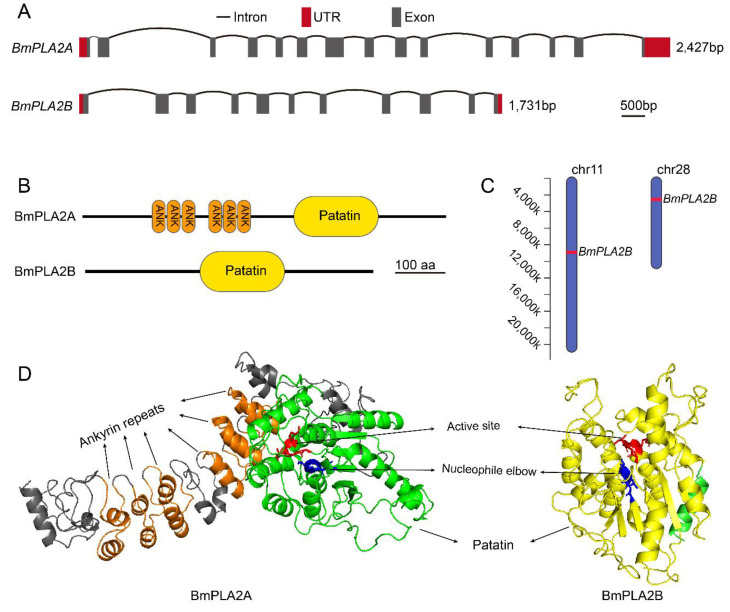
Two *calcium-independent phospholipase A2 (iPLA2)* genes from *Bombyx mori*. (**A**) The gene structure of *BmiPLA2A* and *BmiPLA2B* in silkworm. Exons and introns are represented by black line segments and grey boxes, and 5′ untranslated regions (UTR, left) and 3′ UTR (right) were shown in red boxes. The black scale bar indicates 500 bp. (**B**) Conserved domains of BmiPLA2A and BmiPLA2B predicted by SMART program. (**C**) The chromosome location of *BmiPLA2A* and *BmiPLA2B*. (**D**) Three-dimensional structure prediction of BmiPLA2A and BmiPLA2B. The six ankyrin repeats of BmiPLA2A are colored with orange. The conserved patatin domains of BmiPLA2A and BmiPLA2B were colored with green and yellow, respectively. The active sites and nucleophile elbows were shown in red and blue in the sticks, respectively.

In order to identify the genes of interest in the silkworm, *B. mori* genome, we performed multiple sequence searches using BLAST analysis at the National Center for Biotechnology Information (NCBI) database. By conducting multiple searches, we found two PLA2 sequences from the *B. mori,* which were identified and named as *BmiPLA2A* and *BmiPLA2B.* The obtained sequences were confirmed by using PCR cloning and sequencing. The *BmiPLA2A* included a 2427 base-pair open reading frame (ORF) (Figure S1) that encoded 808 amino acid residues. *BmiPLA2B,* on the other hand, comprised 1731 bp (Figure S2), which encoded a protein of 576 amino acids. BmiPLA2A protein has a molecular weight of 88.02 kDa and a theoretical pI of 6.59. The BmiPLA2B protein comprises a molecular weight of 63.87 kDa and a theoretical pI of 9.64. In addition, both of these genes are located on different chromosomes and have distinct gene architectures. *BmiPLA2A* is located on chromosome 11 in the silkworm genome, with 15 exons and 14 introns, whereas *BmiPLA2B* is located on chromosome 28, with 11 exons and 10 introns (Figure 1). They have certain similarities in amino acid sequences, including patatin domain, motifs of nucleophile elbow, and active site (Figure 1B,D, Figures S1 and S2). While BmiPLA2B shares several characteristics with BmiPLA2A, BmiPLA2B does not contain ankyrin repeats, which are composed of six ANK domains.

#### 3.1.2. Conserved Analysis and Phylogenetic Analysis of BmiPLA2s Protein in Silkworm

**Figure 2:**
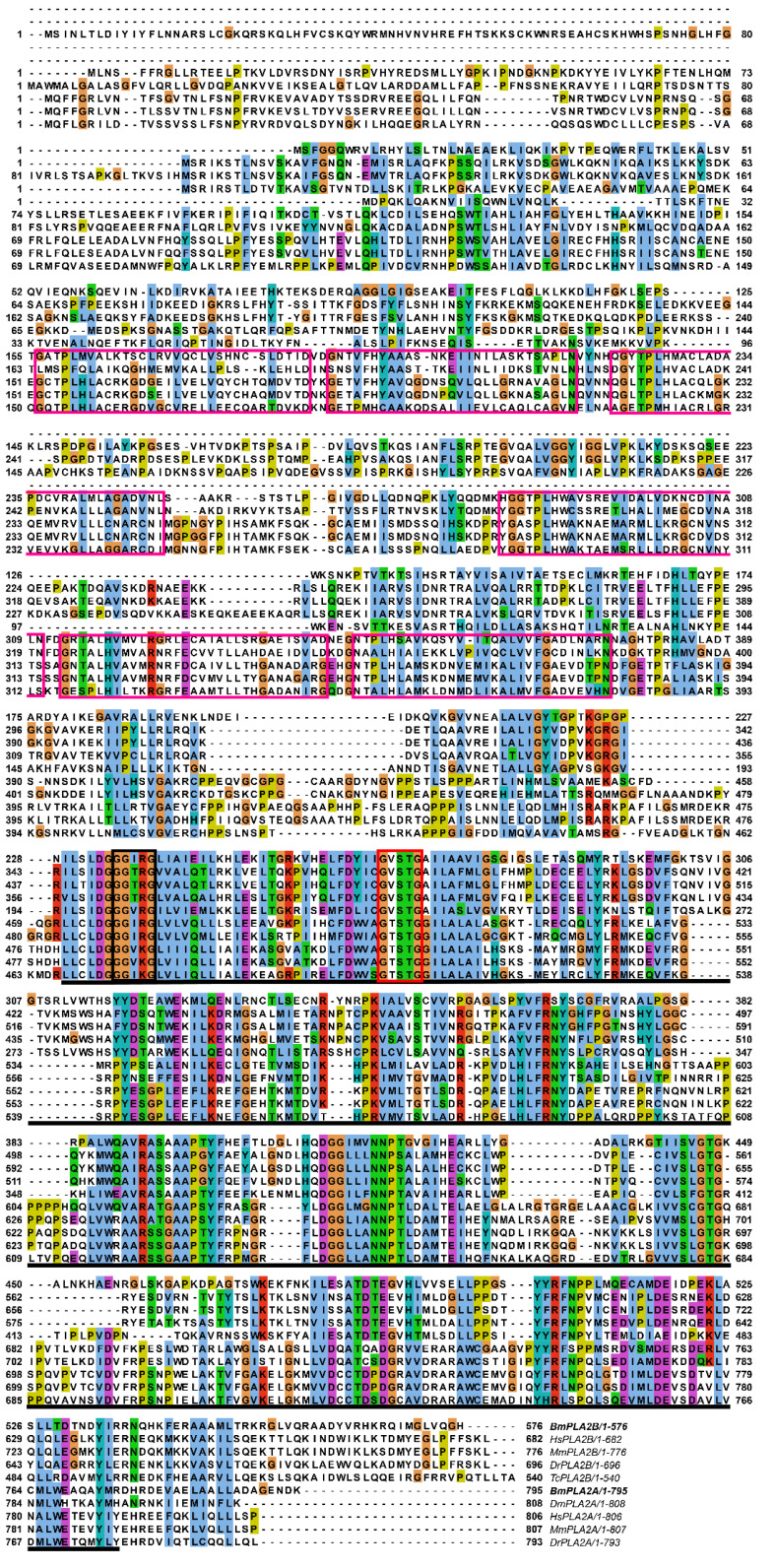
Alignment of BmiPLA2A and BmiPLA2B protein with other homologous proteins. The conserved active site “GGXRG” was marked with a black rectangle, and the conserved nucleophile elbow “GXSTG” was marked with a red rectangle. GenBank accession numbers are as follows: Calcium-independent phospholipase A2 from *Drosophila melanogaster* (NP_729565.2), *Homo sapiens* (NP_001336793.1), *Mus musculus* (NP_001185952.1), *Danio rerio* (NP_998262.1), *Tribolium castaneum* (EFA06489.1), *Homo sapiens* (NP_001242939.1), *Mus musculus* (NP_080440.2), and *Danio rerio* (XP_001918731.2).

In order to identify conserved domains and motifs architecture, multiple sequence alignments were carried out by comparing amino acid sequences of the iPLA2A and iPLA2B proteins from both vertebrates and invertebrates. Homologous sequence alignments revealed that iPLA2As from a wide range of taxa shared highly conserved posterior regions where they contained a patatin domain and a comparatively less conserved frontier region containing six ankyrin repeats that were connected by a short unconserved middle region (Figure S3). IPLA2Bs are highly conserved in the middle and posterior regions, and the patatin domains in the posterior regions are less conserved (Figure S4). In addition, by multiple sequence alignments, we found that active site “GGxRG” and a lipase signature motif “GxSTG” are conserved in both groups (Figure 2).

**Figure 3:**
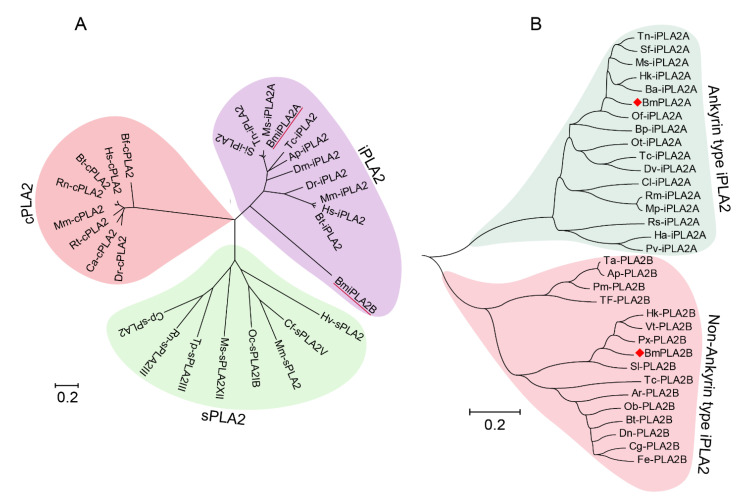
Phylogenetic analysis of BmiPLA2A and BmiPLA2B. (**A**) Phylogenetic analysis of BmiPLA2A and BmiPLA2B (underlined in red) with other iPLA2, cellular PLA2 (cPLA2) and secretory PLA2 (sPLA2) from different species. (**B**) Phylogenetic analysis of two subgroups of insect iPLA2s. BmiPLA2A and BmiPLA2B are labeled with red diamond. The organisms and GenBank accession numbers are listed as follows: Calcium-independent phospholipase A2 from *Homo sapiens* (AAD30424.1), Mus musculus (NP_001185954.1), *Acyrthosiphon pisum* (XP_001944054.1), *Bos taurus* (XP_024848555.1), *Drosophila melanogaster* (NP_729567.2), *Tribolium castaneum* (XP_015833132.1), *Manduca sexta* (XP_030021740.1), *Spodoptera litura* (XP_022829935.1), *Trichoplusia ni* (XP_026746517.1), *Danio rerio* (NP_998262.1), *Aptenodytes patagonicus* (KAF1649516.1), *Athalia rosae* (XP_012263482.1), *Bombus terrestris* (XP_012166304.1), *Colletes gigas* (XP_043250117.1), *Dufourea novaeangliae* (KZC12673.1), *Formica exsecta* (XP_029667145.1), *Formica exsecta* (XP_029667146.1), *Hyposmocoma kahamanoa* (XP_026316138.1), *Osmia bicornis* (XP_029043715.1), *Plutella xylostella* (XP_011550190.2), *Protobothrops mucrosquamatus* (XP_015679970.1), *Spodoptera litura* (XP_022837497.1), *Tachysurus fulvidraco* (XP_026996720.1), *Tribolium castaneum* (EFA06489.1), *Tyto alba* (XP_042648402.1), *Vanessa tameamea* (XP_026499977.1), *Bicyclus anynana* (XP_023941666.1), *Bombus pyrosoma* (XP_043599526.1), Cimex lectularius (XP_024082546.1), *Diabrotica virgifera virgifera* (XP_028131355.1), *Helicoverpa armigera* (XP_021198040.1), *Hyposmocoma kahamanoa* (XP_026317005.1), *Manduca sexta* (XP_030021740.1), *Myzus persicae* (XP_022176520.1), *Onthophagus taurus* (XP_022912622.1), *Ostrinia furnacalis* (XP_028157864.1), *Penaeus vannamei* (XP_027210009.1), *Rhipicephalus sanguineus* (XP_037523510.1), *Rhopalosiphum maidis* (XP_026822162.1), *Spodoptera frugiperda* (XP_035445232.1), *Tribolium castaneum* (XP_008196222.1), *Trichoplusia ni* (XP_026746517.1); Cytosolic phospholipase A2 from *Homo sapiens* (NP_077734.2), *Mus musculus* (NP_032895.1), *Bos taurus* (NP_001069332.1), *Rattus norvegicus* (NP_598235.2), *Danio rerio* (XP_017208191.1), *Rana temporaria* (XP_040215687.1), *Branchiostoma floridae* (XP_035685090.1), *Carassius auratus* (XP_026139465.1); Secretory Phospholipase A2 from *Mus musculus* (AAF22290.1), *Culex quinquefasciatus* (EDS35072.1), *Manduca sexta* (XP_030040460.1,sPLA2GXII), *Rattus norvegicus* (NP_001099485.1,sPLA2GIII), *Culex quinquefasciatus* (EDS36384.1,sPLA2), *Oryctolagus cuniculus* (Q7M334.1,sPLA2GIB), *Trichinella pseudospiralis* (KRZ32035.1,sPLA2GIII), *Camelus ferus* (EQB78640.1,sPLA2GV), and *Metarhizium acridum* (EFY85256.1,sPLA2GXIII).

Evolutionary analysis showed that PLA2s formed three separate branches, including cPLA2, sPLA2, and iPLA2, with BmiPLA2A and BmiPLA2B forming a distinct clade with the iPLA2 group (Figure 3A). Another phylogenetic tree was constructed to further understand the evolutionary differences between BmiPLA2A and BmiPLA2B. Interestingly, according to the presence of the N-terminal ankyrin repeat domain, iPLA2 is divided into two distinct branches, which is a unique feature of this gene. BmiPLA2A was separated into ankyrin type, and BmiPLA2B was separated into non-ankyrin type (Figure 3B).

#### 3.1.3. Tissue Distribution of BmiPLA2s

**Figure 4:**
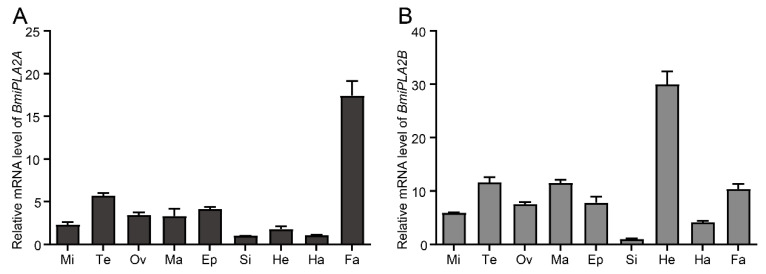
Tissue distribution of *BmiPLA2A* (**A**) and *BmiPLA2B* (**B**). Si: silk gland, Ma: Malpighian tube, Ep: epidermis, Fa: fat body, He: head, Te: testis, Mi: midgut, and Ha: hemocyte, and Ov: Ovary. The expression levels in the silk gland were used as the calibrator for each group.

*BmiPLA2A* and *BmiPLA2B* expression levels in various tissues of silkworm larvae were evaluated using qRT-PCR assay (Figure 4). The ubiquitous transcription levels of *BmiPLA2A* and *BmiPLA2B* were observed in all the tested tissues, including hemocyte, testis, epidermis, head, Malpighian tubule, midgut, silk gland, fat body, and ovary. However, their tissue distribution patterns differed except for their relatively lowest transcription levels in the silk gland. *BmiPLA2A* produced the highest mRNA expression levels in the fat body, followed by moderate levels in the testis and epidermis. In contrast, the lowest levels were observed in the silk gland and hemocytes (Figure 4A). *BmiPLA2B* mRNAs were most abundant in the head, followed by the testis, Malpighian tubule, and fat body, with the lowest mRNA expression levels found in the hemocyte and silk gland.

#### 3.1.4. Temporal Expression Profiles of BmiPLA2s in Silkworm

**Figure 5:**
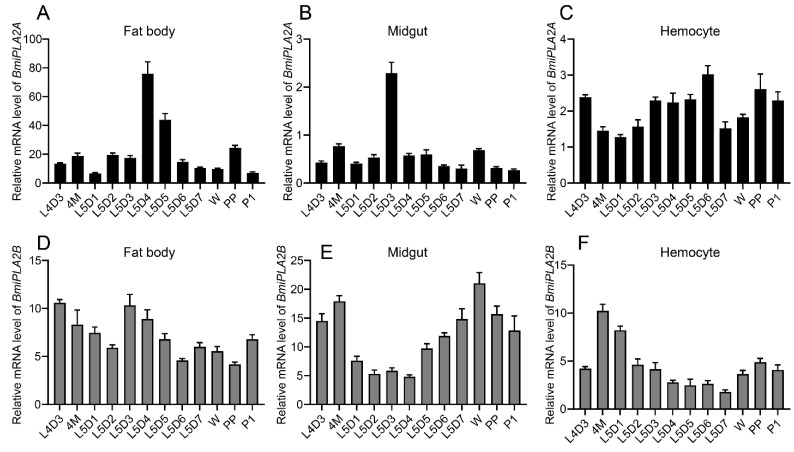
The temporal expression profiles *BmiPLA2A* and *BmiPLA2B* in the fat body (**A**,**D**), midgut (**B**,**E**), and hemocyte (**C**,**F**) from fourth instar day 3 (L4D3) to the pupal stage. 4M: fourth molting stage, W: wandering stage, PP: pre-pupa stage, P1: day 1 of pupa stage.

In order to investigate the temporal expression profiles of *BmiPLA2A* and *BmiPLA2B,* fat body, midgut, and hemocytes from larvae at fourth instar day one through pupae were collected. The results revealed that the mRNA expression of *BmiPLA2A* and *BmiPLA2B* had different expression patterns on the tested *B. mori* stages (Figure 5). This inconsistency was evident since the expression of *BmiPLA2A* was much higher in fat bodies when compared to the expression of *BmiPLA2B* (Figure 5A,D). In the fat body, *BmiPLA2A* had the maximum expression levels at the fifth instar at 4 days, and then the expression levels gradually decreased, reaching the lowest levels at the wandering stage. *BmiPLA2B* had comparably low expression levels at L5D2 and L5D6 to the pre-pupa stage. In the midgut, *BmiPLA2A* had a moderate expression peak during the fourth molting stage and wandering stage, and the highest expression level appeared at L5D3; the expression level of *BmiPLA2B* showed a decrease-to-increase trend with the highest mRNA levels appearing at the fourth molting stage and wandering stage (Figure 5B,E). In the hemocytes, *BmiPLA2A* showed relatively low expression levels near the fourth molting and wandering stages, whereas *BmiPLA2B* had the highest transcription level in the fourth molting stage and a moderate peak level at the pre-pupa stage (Figure 5C,F).

**Figure 6:**
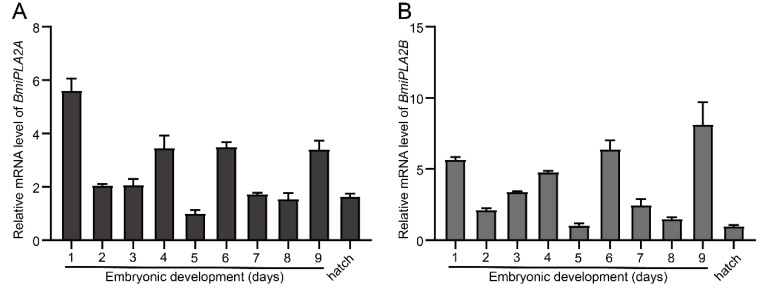
The temporal expression profiles *BmiPLA2A* (**A**) and *BmiPLA2B* (**B**) in embryonic developmental stages.

In addition, total RNA was isolated from embryonic developmental stages and hatch day to analyze the embryonic developmental profile of *BmiPLA2*s, and then total RNA was reverse transcribed into cDNA. The results of qRT-PCR showed that the expression levels of *BmiPLA2A* and *BmiPLA2B* followed mRNA expression patterns that were quite similar. Furthermore, we observed that *BmiPLA2*s mRNA levels were at their peak in the middle stages of embryonic development (Figure 6).

#### 3.1.5. 20-Hydroxyecdysone Enhanced the Expression Levels of BmiPLA2s

**Figure 7:**
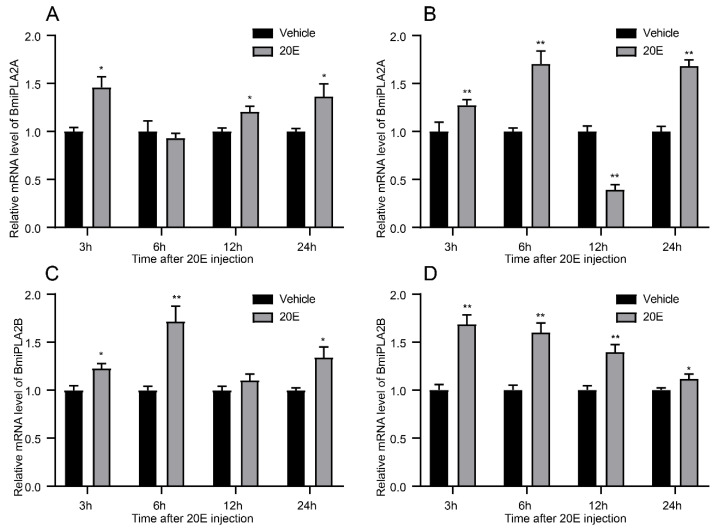
Expression of *BmiPLA2A* in hemocytes (**A**) and epidermis (**B**) of 20-E (20-hydroxyecdysone) induced silkworm larvae, and expression of *BmiPLA2B* in hemocytes (**C**) and epidermis (**D**) of 20-E (20-hydroxyecdysone) induced silkworm larvae. The asterisks indicate the significance was statistically significant or extremely significant (* *p* < 0.05, ** *p* < 0.01 and *** *p* < 0.001).

In order to examine the expression patterns of *BmiPLA2s* response to ecdysone, silkworm larvae were administrated with 20-hydroxyecdysone, a steroid hormone. The results revealed that 20-hydroxyecdysone significantly increased the expression level of *BmiPLA2s* in general (Figure 7). In both hemocytes (Figure 7A) and the epidermis (Figure 7B), *BmiPLA2A* mRNA levels induced by 20-hydroxyecdysone showed an upward-downward-upward trend, and the mRNA level of *BmiPLA2A* at 12 h was significantly decreased by 20-hydroxyecdysone in the epidermis (Figure 7B). The transcription levels of *BmiPLA2B* in hemocyte and epidermis gradually increased and then declined after exposure to 20-hydroxyecdysone treatment, with peak levels observed at 6 h in hemocytes and 3 h in the epidermis (Figure 7C,D).

#### 3.1.6. Responses of BmiPLA2s to Microbial Challenges

**Figure 8:**
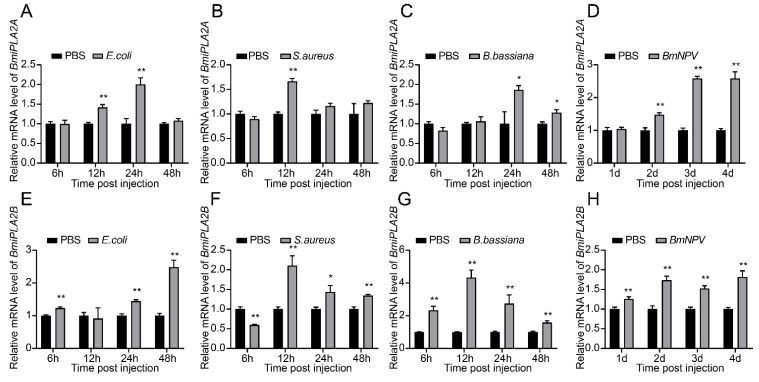
Temporal mRNA expression of *BmiPLA2A* and *BmiPLA2B* in hemocytes of immune challenged silkworm larvae. The expression profiles at 6, 12, 24, and 48 h were analyzed after treatment with (**A**,**E**) Gram-negative bacteria *Escherichia coli* (*E. coli*), (**B**,**F**) Gram-positive bacteria *Staphylococcus aureus* (*S. aureus*), (**C**,**G**) fungus *Beauveria bassiana* (*B. bassiana*), and (**D**,**H**) virus *Bombyx mori nucleopolyhedrovirus* (*BmNPV*). The asterisks indicate the significance was statistically significant or extremely significant (* *p* < 0.05, ** *p* < 0.01 and *** *p* < 0.001).

**Figure 9:**
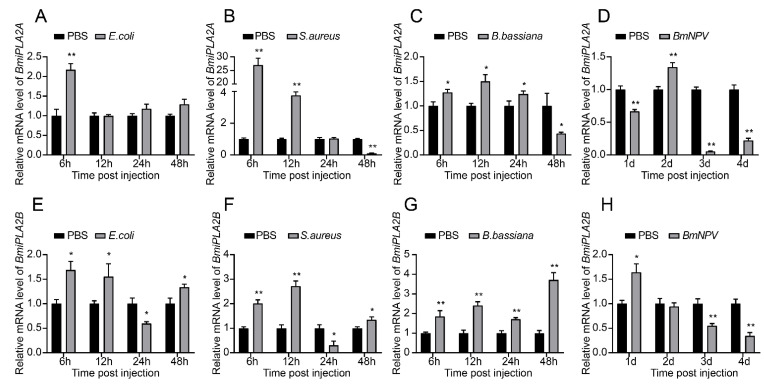
Temporal mRNA expression of *BmiPLA2A* and *BmiPLA2B* in fat bodies of immune challenged silkworm larvae. The expression profiles at 6, 12, 24, and 48 h were analyzed after treatment with (**A**,**E**) Gram-negative bacteria *Escherichia coli* (*E. coli*), (**B**,**F**) Gram-positive bacteria *Staphylococcus aureus* (*S. aureus*), (**C**,**G**) fungus *Beauveria bassiana* (*B. bassiana*), and (**D**,**H**) virus *Bombyx mori nucleopolyhedrovirus* (*BmNPV*). The asterisks indicate the significance was statistically significant or extremely significant (* *p* < 0.05, ** *p* < 0.01 and *** *p* < 0.001).

We performed a qRT-PCR assay to determine relative expression patterns of *BmiPLA2s* in two immune-related tissues (hemocyte and fat body) after infection with *E. coli*, *S. aureus*, *B. bassiana*, or *BmNPV*. As shown in Figure 8 and Figure 9, the mRNA expression levels of *BmiPLA2s* were significantly changed with different degrees in response to immunological stress. Furthermore, *BmiPLA2*s expression levels in hemocytes and fat bodies varied with the type of pathogen used, and the maximum expression level induced differed at different time points in the examined tissues. When larvae were exposed to immunological challenges involving *E. coli*, *S. aureus*, or *B. bassiana*, the expression levels gradually or sharply increased at first and then decreased to some extent. Of note, when exposed to *E. coli* infection, the expression levels of *BmiPLA2A* in the fat body increased dramatically at 3 h post-infection and then returned to normal after that (Figure 9A), while the expression levels of *BmiPLA2B* continuously increased and reached a peak at 48 h (Figure 8E). Moreover, among the four tested pathogens, *S. aureus* strongly induced the transcription levels of *BmiPLA2A* in the fat body. However, although *BmNPV* stimulation increased mRNA levels of *BmiPLA2A* and *BmiPLA2B* in hemocytes (Figure 8D,H) and in the fat body, *BmNPV* mediated immune stress significantly increased the expression levels of *BmiPLA2A* and *BmiPLA2B* in the first two days and dropped down lower than normal levels in the following time (Figure 9D,H).

## 4. Discussion

PLA2s are an important group of a gene family, serving as a crucial regulator of diverse physiological processes, including development and immunity [24]. *sPLA2s* have been described in a variety of insect species [10,11,12,14,16]. However, only a few *iPLA2s* have been identified among insects, such as *Spodoptera exigua* [7,8,9] and *Drosophila melanogaster* [10]. The present study reports two novel *iPLA2*s from *B. mori,* and their expression patterns. With the exception of the comparable C-terminal lipase catalytic domain, there are numerous differences in their putative protein sequences. For example, the N-terminal region of BmiPLA2A has a unique ankyrin repeat domain that serves as a scaffold for protein–protein interactions [25]. BmiPLA2A (GTSTG) differs from BmiPLA2B (GVSTG) in that it has a distinct lipase motif. Likewise, *Spodoptera exigua*, SeiPLA2A, and SeiPLA2B have the same protein sequence differences [9]. Due to the greatest degree of similarity between the protein sequence features of iPLA2s in *B. mori* to SeiPLA2A and SeiPLA2B in *Spodoptera exigua*, they have been classified as BmiPLA2A and BmiPLA2B. Homologous protein sequence alignments revealed that iPLA2As were conserved at both the N-terminus and the C-terminus and that iPLA2Bs were conserved at the C-terminus. Phylogenetic analysis showed that BmiPLA2s were clustered with the iPLA2 group and that they were further subdivided into two distinct groups. Collectively, we inferred that *BmiPLA2A* and *BmiPLA2B* are two members of the *iPLA2* family who may play multiple biological roles in silkworm development and immunity.

Tissue distribution patterns of genes are an important parameter for determining their possible biological roles. Therefore, in the present study, first, we analyzed the tissue expression profiles iPLA2 in *B. mori.* In *Spodoptera exigua*, *PLA2A* is expressed strongly in various tissues, with approximately similar expression patterns [7]. However, in *B. mori* tissues, the expression levels of *BmiPLA2A* varied greatly. For example, the mRNA level of *BmiPLA2A* in the fat body was nearly 17-fold higher than that in the silk gland. *BmiPLA2B* is also widely transcribed in all tested tissues, with the silk gland having the lowest transcription level, which is consistent with *BmiPLA2A*. This expression is not consistent with *BmiPLA2B* homolog *SeiPLA2B* of *Spodoptera exigua*, which is not expressed in the digestive tract [8]. In addition, *BmiPLA2*s were found to be expressed at various developmental stages in the three immune-related tissues of *B. mori*. The broad tissue and temporal distribution of *BmiPLA2s* indicate that they may be involved in various biological functions [9].

A growing body of evidence suggests that this gene family has a crucial biological role in the development of living organisms [12,13,15,26]. Thus, to determine the possible functions of *PLA2*s in development, we quantified the expression levels of these genes after 20-hydroxyecdysone administration. The results revealed that *BmiPLA2A* and *BmiPLA2B* expression significantly increased. In *Spodoptera exigua, Se-sPLA2A* has been reported to be regulated by pyriproxyfen, a juvenile hormone analog [12]. Moreover, a number of studies demonstrate that 20-hydroxyecdysone can play an important role in the metamorphosis process [20,22,27,28]. These results suggest that the *PLA2A* family may have a crucial biological role in the developmental process of insects. However, further studies are needed to verify their functions.

It has been well documented that sPLA2s mediate both cellular and humoral immune responses via the eicosanoid signaling pathway [12,13,16,29]. However, despite the fact that they function as a release of fatty acids hydrolyzed from phospholipids, another subgroup of the *PLA2* gene family, *iPLA2*, is still poorly understood in insect-immune responses [8,30]. Therefore, we quantified the mRNA levels of *iPLA2* genes after the immune challenge. The results showed that viral, bacterial, or fungal pathogen stimuli significantly impacted *BmiPLA2A* and *BmiPLA2B* expression levels. Interestingly, viral infection increased the mRNA levels of *BmiPLA2*s in the fat body in the early infection while suppressing their transcription levels in the fat body at certain times during infection. Previous studies have shown that the expression of *Se-iPLA2A* and *Se-iPLA2B* in *Spodoptera exigua* are induced upon bacterial challenge [9].

*BmiPLA2A* and *BmiPLA2B* may play important biological roles in the immunity of *B. mori,* as evidenced by their broad responses to diverse microbial infections. Insect hemocytes are involved in both cellular (e.g., phagocytosis and encapsulation) and humoral immune responses, including AMPs release [31]. Increased transcription levels of *BmiPLA2A* and *BmiPLA2B* in response to immune challenge may contribute to hemocyte [12,32,33], and fat body also mediated immune responses, such as the synthesis AMPs [34,35]. Therefore, increased expression levels may improve the humoral immune responses via lipid homeostasis. However, further studies are required to elucidate the biological mechanisms of *iPLA2s* in the immunity of silkworm.

Taken together, this study reports two novel iPLA2 genes that may play a vital role in the development and immunity of silkworm. In *B. mori,* the tissue distribution and temporal expression profiles of identified *iPLA2s* were examined. The mRNA levels of both the genes were increased by 20-hydroxyecdysone induction. The immune challenge with various types of pathogens induced the expression levels of *BmiPLA2A* and *BmiPLA2B* in immune-related tissues. This study broadens our understanding of *iPLA2s* in insect immunity and development, providing a baseline for future studies on insect development and immune responses.

## Figures and Tables

**Table 1:** Primers used in this study.

Gene Name and Purpose	Primer Name	Annealing Temperature (°C)	Sequence (5’–3’)
*BmiPLA2A* cDNA cloning	BmiPLA2AF	54.36	ATGCTGAATTCATTTTTTCGTG
	BmiPLA2AR	56.74	CTAACCGGTCGTGCCGTTGTGTTC
*BmiPLA2B* cDNA cloning	BmiPLA2BF	54.47	AGCCAGGCTACTATACGGAGCG
	BmiPLA2BR	57.76	TCAGTGCCCCTGGACTAGGCCCATG
*BmiPLA2A* qPCR	iPLA2AF	55.40	ACTCATGCGGCGGTAAAGAA
	iPLA2AR	57.45	TCTCCTTGTTGCTAGCTGCC
*BmiPLA2B* qPCR	iPLA2BF	57.45	TCGGAGCCAAGTTGGAAGTC
	iPLA2BR	57.45	CTGACCGCACCTTCCTTGAT
*Sw22934* qPCR	sw22934 F	55.40	TTCGTACTGGCTCTTCTCGT
	sw22934 R	51.95	CAAAGTTGATAGCAATTCCCT

## Supplementary Materials

The following are available online at https://www.mdpi.com/article/10.3390/cimb44020054/s1, Figure S1: Nucleotide and amino acid sequence of the *Bombyx mori BmiPLA2A* gene. Figure S2: Nucleotide and amino acid sequence of the *Bombyx mori BmiPLA2B* gene. Figure S3: Alignment of the BmiPLA2A protein with other homologous proteins. Figure S4: Alignment of the BmiPLA2B protein with other homologous proteins.
