# Supplementary material for: Molecular Characterization of Two Genes Encoding Novel Ca2+-Independent Phospholipase A2s from the Silkworm, Bombyx mori
Source: Curr Issues Mol Biol. 2022 Feb 4;44(2):777–90. doi: 10.3390/cimb44020054 (PMC8929031; doi:10.3390/cimb44020054)
Supplement: Supplementary file 1 [file cimb-44-00054-s001.zip › Figure legend for Suplementary figure.pdf]

Figure S1. Nucleotide and amino acid sequence of the *Bombyx mori BmiPLA2A* gene. The putative patatin domain is marked with green. red, and the active site “GGIR” and the nucleophile elbow “GTSTG” are labeled with a red box and a blue box, respectively. The stop codon is represented by an asterisk.

Figure S2. Nucleotide and amino acid sequence of the *Bombyx mori BmiPLA2B* gene. The putative patatin domain is marked with yellow. The active site “GGIR” and the nucleophile elbow “GVSTG” are labeled with a red box and a blue box, respectively. The stop codon is represented by an asterisk.

Figure S3. Alignment of the BmiPLA2A protein with other homologous proteins. Six ankyrin repeats were marked with black triangles. The active site “GGIR” and the nucleophile elbow “GVSTG” are labeled with a black box and a red box, respectively. The conserved patatin domains are underlined. The GenBank accession numbers are listed as follows: *Drosophila melanogaster* (NP_729565.2), *Homo sapiens* (NP_001336793.1), *Mus musculus* (NP_001185952.1), *Danio rerio* (NP_998262.1).

Figure S4. Alignment of the BmiPLA2B protein with other homologous proteins. The active site “GGIR” and the nucleophile elbow “GVSTG” are labeled with a black box and a red box, respectively. The conserved patatin domains are underlined. The GenBank accession numbers are listed as follows: *Tribolium castaneum* (EFA06489.1), *Homo sapiens* (NP_001242939.1), *Mus musculus* (NP_080440.2), *Danio rerio* (XP_001918731.2)
