# Supplementary figures and images for: Molecular Characterization of Two Genes Encoding Novel Ca2+-Independent Phospholipase A2s from the Silkworm, Bombyx mori
Source: Curr Issues Mol Biol. 2022 Feb 4;44(2):777–90. doi: 10.3390/cimb44020054 (PMC8929031; doi:10.3390/cimb44020054)

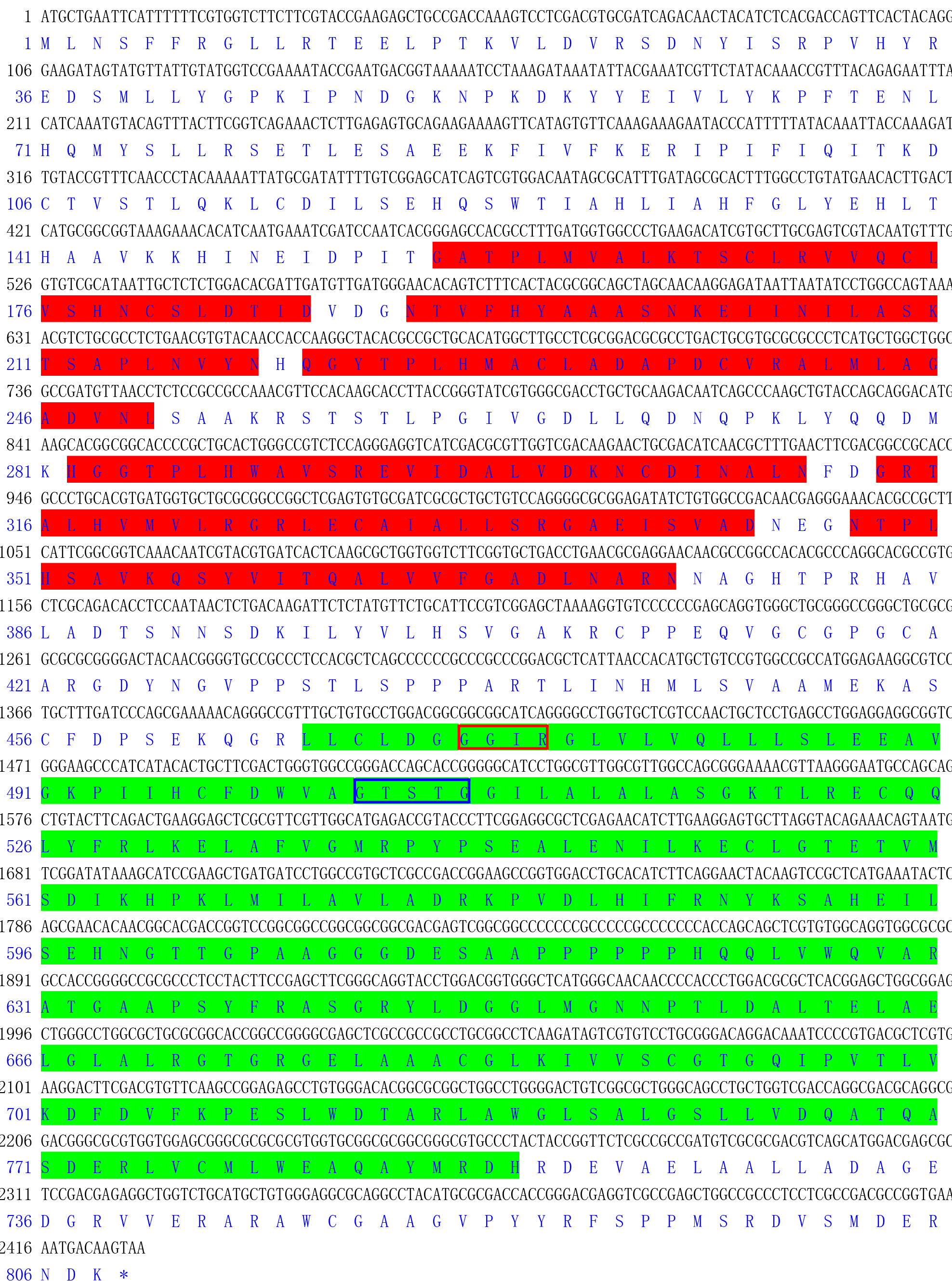

Supplement: Supplementary file 1 [file cimb-44-00054-s001.zip › Figure S1.tif]

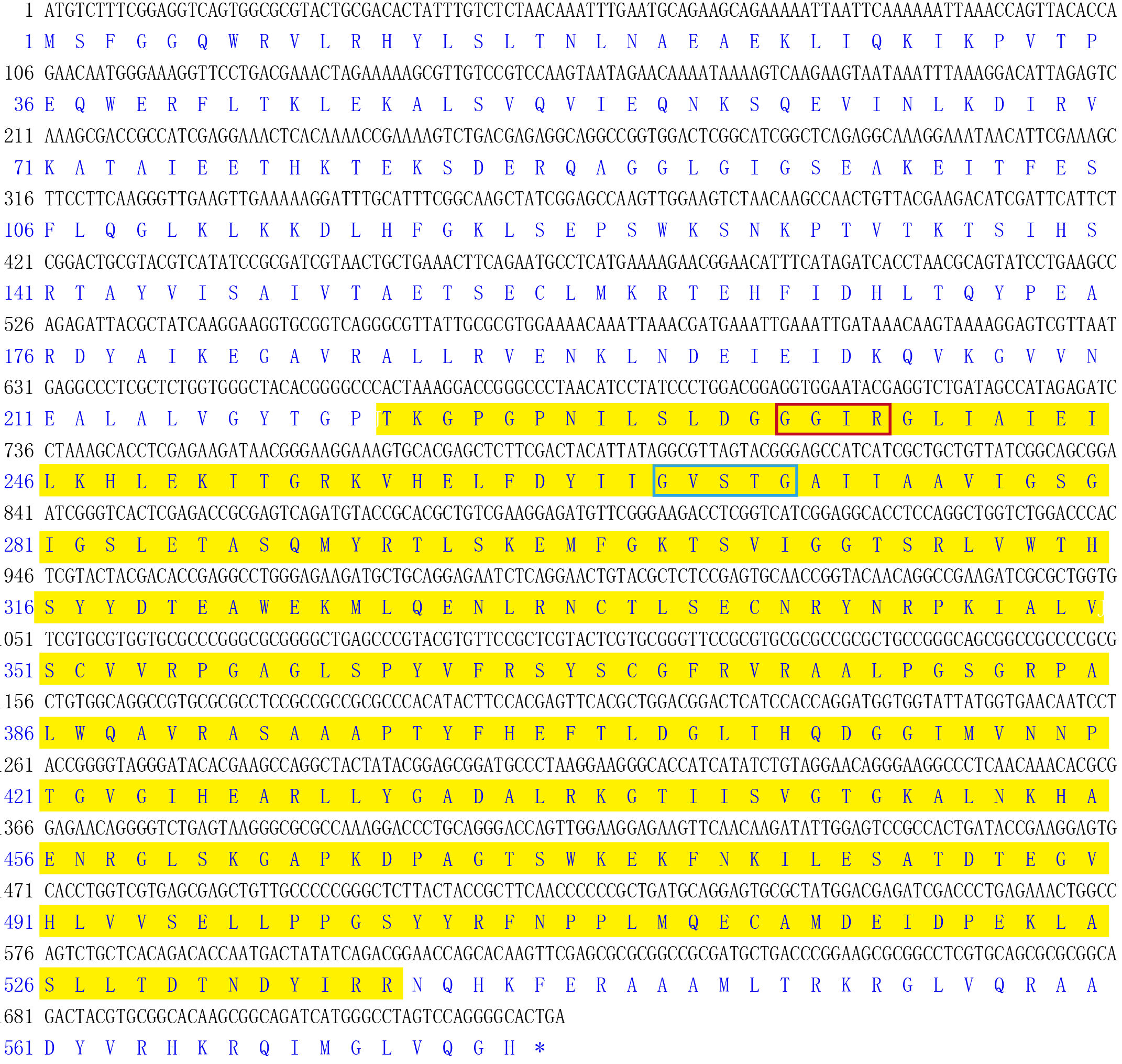

Supplement: Supplementary file 1 [file cimb-44-00054-s001.zip › Figure S2.tif]

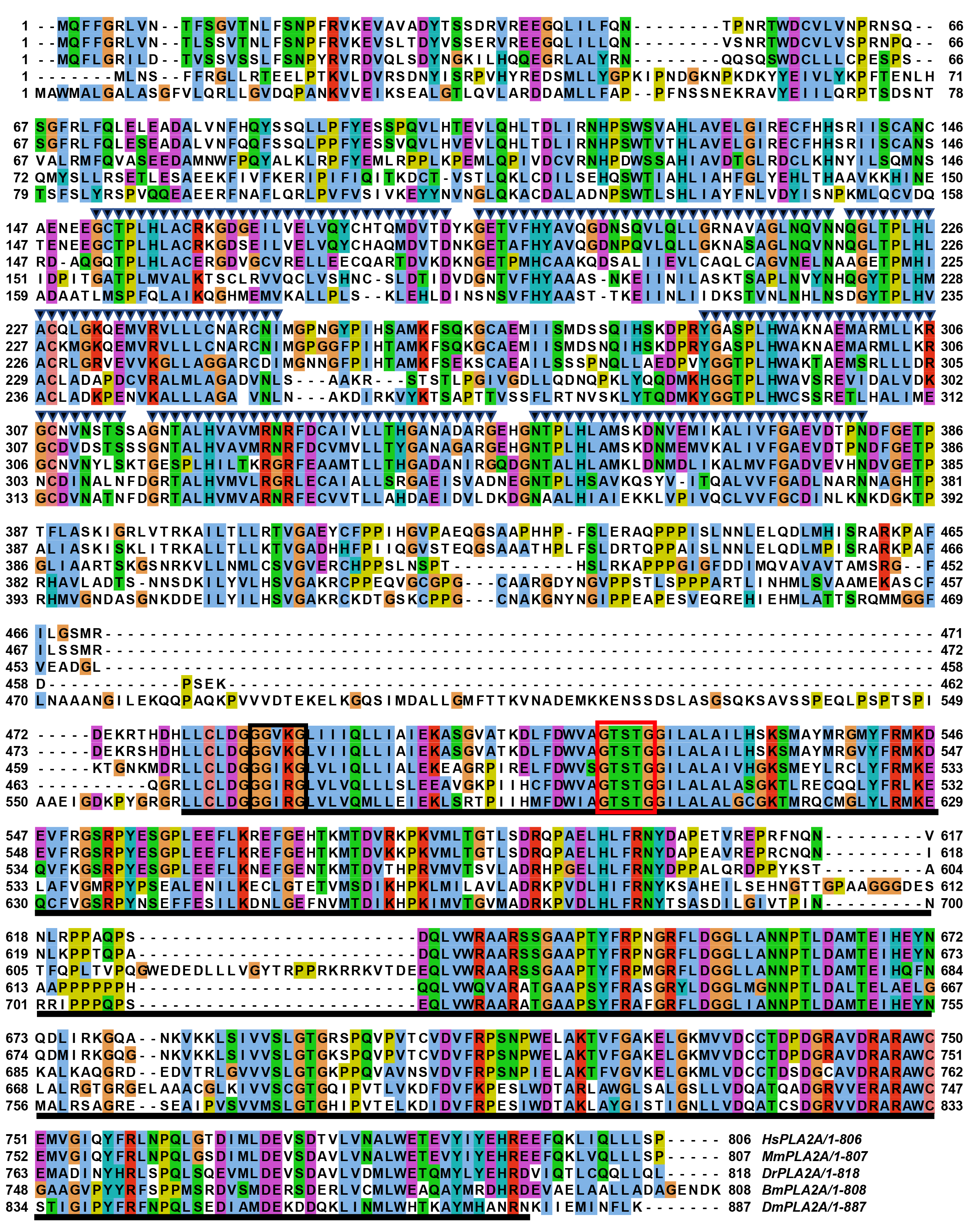

Supplement: Supplementary file 1 [file cimb-44-00054-s001.zip › Figure S3.tif]

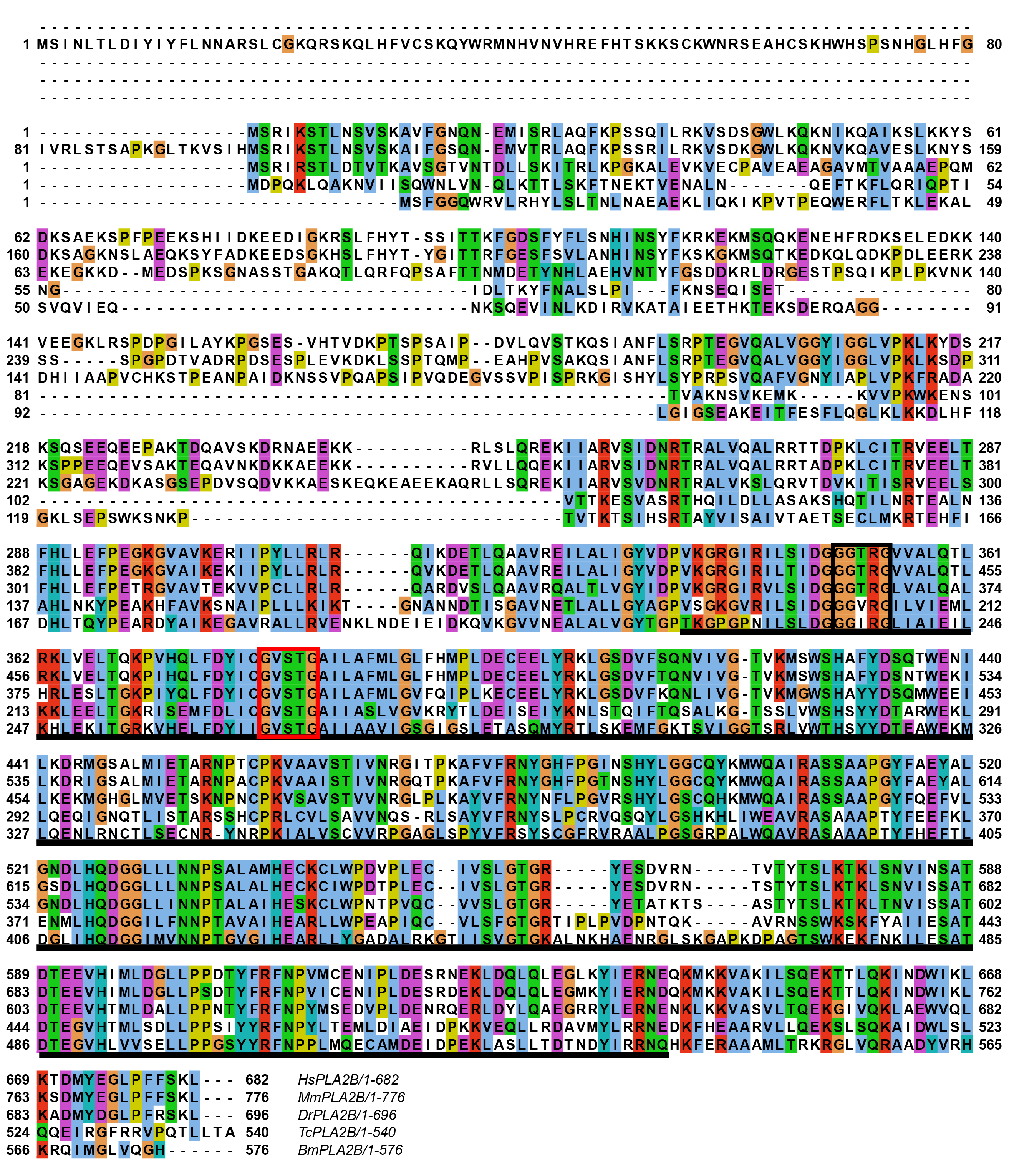

Supplement: Supplementary file 1 [file cimb-44-00054-s001.zip › Figure S4.tif]
